# Climate, soil type, and geographic distribution of actinomycetoma cases in Northeast Mexico: A cross-sectional study

**DOI:** 10.1371/journal.pone.0232556

**Published:** 2020-05-08

**Authors:** Jesus Alberto Cardenas-de la Garza, Oliverio Welsh, Adrian Cuellar-Barboza, Karina Paola Suarez-Sanchez, Luis Gerardo Cruz-Gomez, Estephania De la Cruz-Valadez, Jorge Ocampo-Candiani, Lucio Vera-Cabrera

**Affiliations:** Servicio de Dermatología, Facultad de Medicina y Hospital Universitario, Universidad Autónoma de Nuevo León, Monterrey, Mexico; Faculty of Science, Ain Shams University (ASU), EGYPT

## Abstract

**Background:**

Mycetoma is a chronic, granulomatous infection of subcutaneous tissue, that may involve deep structures and bone. It can be caused by bacteria (actinomycetoma) or fungi (eumycetoma). There is an epidemiological association between mycetoma and the environment, including rainfall, temperature and humidity but there are still many knowledge gaps in the identification of the natural habitat of actinomycetes, their primary reservoir, and their precise geographical distribution. Knowing the potential distribution of this infection and its ecological niche in endemic areas is relevant to determine disease management strategies and etiological agent habitat or reservoirs.

**Methodology/principal findings:**

This was an ambispective descriptive study of 31 patients with actinomycetoma. We determined the biophysical characteristics including temperature, precipitation, soil type, vegetation, etiological agents, and mapped actinomycetoma cases in Northeast Mexico. We identified two disease cluster areas. One in Nuevo Leon, with a predominantly kastanozems soil type, with a mean annual temperature of 22°, and a mean annual precipitation of 585.2 mm. Herein, mycetoma cases were produced by *Actinomadura pelletieri*, *Actinomadura madurae*, *Nocardia brasiliensi*s, and *Nocardia* spp. The second cluster was in San Luis Potosí, where lithosols soil type predominates, with a mean annual temperature of 23.5° and a mean annual precipitation of 635.4 mm. In this area, all the cases were caused by *N*. *brasiliensis*. *A*. *madurae* cases were identified in rendzinas, kastanozems, vertisols, and lithosols soils, and *A*. *pelletieri* cases in xerosols, kastanozems, and rendzinas soils. Previous thorn trauma with *Acacia* or *Prosopis* plants was referred by 35.4% of subjects. In these states, the presence of thorny plants, such as *Acacia* spp., *Prosopis* spp., *Senegalia greggi*, *Vachellia farnesiana* and *Vachellia rigidula*, are common.

**Conclusions/significance:**

Mapping this neglected tropical infection aids in the detection of disease cluster areas, the development of public health strategies for early diagnosis and disease prediction models; this paves the way for more ecological niche etiological agent research.

## Introduction

Mycetoma is a chronic, granulomatous infection of subcutaneous tissue, which can involve deep structures and bone. It most commonly affects the foot or hand causing disability in late stages [1, 2]. Mycetoma is caused by a myriad of microorganisms including bacteria (actinomycetoma) and fungi (eumycetoma). The most prevalent etiological agents of actinomycetoma are *N*. *brasiliensis*, *A*. *madurae*, *A*. *pelletieri* and *Streptomyces somaliensis*. Eumycetoma is predominantly caused by *Madurella mycetomatis*, *Trematosphaeria grisea*, and *Scedosporium boydii* [3].

Mycetoma was recognized by the World Health Organization as a Neglected Tropical Disease in 2016 [2]. This disease is more common around the “mycetoma belt” between the latitudes of 15°S and 30°N that includes countries such as Sudan, Mexico, and Senegal [4]. There is an epidemiological association with the environment, including rainfall, temperature and humidity, but there are still many knowledge gaps in the identification of the natural habitat of the causative organisms, their primary reservoir, and their precise geographical distribution [1, 3]. Samy et al. [4], recognizing the knowledge gap regarding the epidemiology and transmission cycle of the causative agents, performed an ecological niche model to map risk of mycetoma infection in Sudan and South Sudan. The model identified a specific area where mycetoma predominates, found the possibility of a mycetoma-*Acacia* association, and provided steps to a robust predictive risk map for the disease. This study represented a landmark in mycetoma epidemiology research. It also shed light on the importance of mapping and identifying soil, climate, and vegetation characteristics on endemic regions.

Mapping mycetoma is important to identify the geographical distribution of cases, disease clusters, and develop disease prediction models. Furthermore, knowing the potential distribution of this infection and its ecological niche in an endemic area is relevant to determine disease management strategies and etiological agent habitats or reservoirs. Mycetoma geographical and environmental information, particularly of actinomycetoma, is limited. We aimed to determine the environmental characteristics including temperature, precipitation, soil type, vegetation, and etiological agent, and map actinomycetoma cases in an endemic region of Northeast Mexico.

## Material and methods

This was an ambispective descriptive study of 31 patients diagnosed with actinomycetoma in the University Hospital “Dr. José Eleuterio González”, a referral hospital in northeast Mexico. A specific and protocolized format is used by the Mycetoma Clinic of the Dermatology Department in our institution. All patients with mycetoma suspicion or diagnosis undergo a structured interview. Data regarding demographics, location of origin, and history of prior trauma or thorn prick (and type of vegetation suspected of causing it) is documented in the patients’ clinical charts. Diagnosis of the etiological agents was made by direct grain examination, histopathology, culture, and/or serology. To diminish bias, only cases with a compatible mycetoma clinical presentation (chronic edema, nodules, fistulae with a serous or purulent discharge) confirmation by these diagnostic methods were included. The location where the mycetoma cases occurred, history of prior trauma, thorn prick, and occupational risk factors were obtained from the clinical files of the department of dermatology from January 2009 to September 2018. Identification to the species level was achieved using nucleotide sequence analysis of a fragment of the small ribosomal subunit (16S) gene [5]. The species in the 31 cases were identified as follows: 20 by culture and nucleotide sequence analysis, 8 by Anti-*N*. *brasiliensis* antibodies, and 3 by direct mycological examination of grains.

Temperature and precipitation were retrieved from governmental climate database from climate stations operating since 1925 to date from Mexico's National Water Commission (CONAGUA). The annual mean of temperature and precipitation from 1925 to 2015 was retrieved from the nearest climate stations of the location where cases occurred for Nuevo Leon, San Luis Potosí, Tamaulipas, Coahuila, Veracruz, and Hidalgo [6]. Presence or absence of Acacia spp, Prosopis spp, Senegalia greggi, Vachellia farnesiana, and Vachellia rigidula, in the states where patients come from, were obtained from the Global Biodiversity Information Facility database (www.gbif.org) [7]. Actinomycetoma occurrences were georeferenced by cities using the coordinates where cases emerged, the 1:50m cultural vector “Admin 1 –States and provinces” shapefile (https://www.naturalearthdata.com/downloads/50m-cultural-vectors/50m-admin-1-states-provinces/) from the Natural Earth database and ArcMap software. We employed the FAO–UNESCO digital soil map of the world (http://www.fao.org/soils-portal/soil-survey/soil-maps-and-databases/faounesco-soil-map-of-the-world/en/) as a reference on the type of soil where the cases occurred [8]. The statistical analysis was performed using IBM SPSS statistical package version 24 (IBM Inc., Armonk, NY, USA). Frequencies were used to describe categorical variables. Variables with normal distribution were described with mean and standard deviation.

### Ethics statement

The protocol was approved by the institutional ethics and research committee (Comité de Ética en Investigación and Comité de Investigación of the University Hospital “Dr. José Eleuterio González”) with reference number of DE17-00009.

## Results

**Fig 1:**
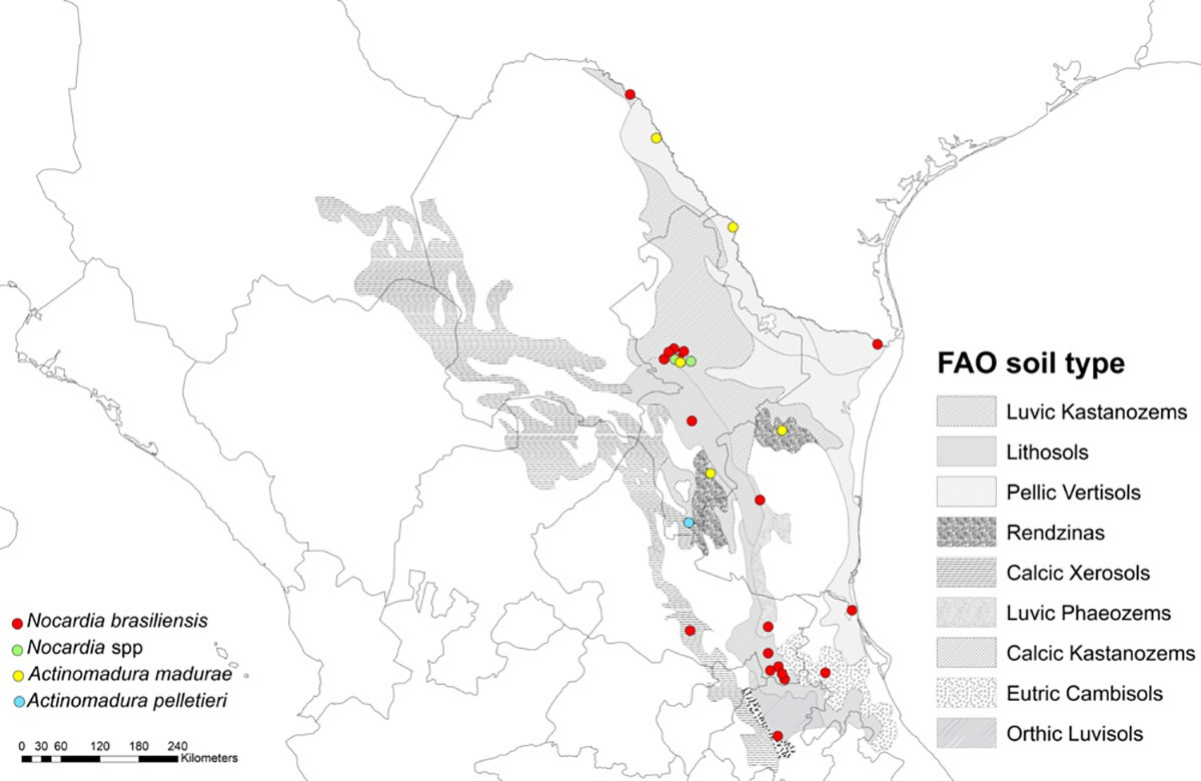
Map of actinomycetoma cases in Northeast Mexico. Figure was created for this publication using a shapefile from the Natural Earth database (naturalearthdata.com) and ArcMap of the ESRI (Environmental Systems Resource Institute) v. 10.5 (Redlands, California). Type of soils per FAO/UNESCO digital soil map of the world.

A total of 31 actinomycetoma cases were included, 24 men (77.4%) and 7 women (22.6%) aged between 15 and 73 years old (median 50). The cases were localized between the latitudes 19° north and 30° north in states where semiarid weather predominates. A total of 13 (41.9%) cases were from Nuevo Leon, 9 (29.0%) from San Luis Potosí, 5 (16.1%) from Tamaulipas, 2 (6.5%) from Coahuila, 1 (3.2%) from Veracruz, and 1 (3.2%) from Hidalgo 1 (Fig 1). Twenty-three patients were from urban areas (74.2%), and 8 from agropastoral areas (25.8%). The main occupation were farmer or agricultural workers in 13 cases (41.9%), construction workers, 6 (19.4%), housewives, 4 (12.9%), and gardener in 1 patient (3.2%). Seven patients had other occupations (22.6%). History of trauma was reported in 12 patients (38.7%), and denied in 10 (32.3%). Six (19.4%) patients did not remember a previous trauma. We could not find information related to history of trauma in 3 (9.7%) patients.

A total of 23 (74.2%) cases were by *N*. *brasiliensis*; 5 (16.1%) were by *A*. *madurae*; 2 (6.5%) by *Nocardia* spp.; and 1 (3.2%) by *A*. *pelletieri*. Complete clinical, diagnostic and therapeutic information of these cases has been reported previously [9]. The mean annual temperature and mean annual precipitation from 1925 to 2015 from the nearest climate stations where cases occurred in Nuevo Leon, San Luis Potosi, Tamaulipas, Coahuila, Veracruz and Hidalgo was obtained as follows, the mean annual temperature ranged from 17.2 to 25.8°C with a mean of 22.9° (SD = 2.07) and a mean annual precipitation of 373.5 to 2713.3 mm with a mean of 1043.4 (SD = 690.6) (Table 1).

**Table 1:** Location, climate, soil type, and etiological agent of mycetoma cases from San Luis Potosí (SLP), Nuevo Leon (NL), Tamaulipas (Tamps), and Coahuila (Coah).

Location	Coordinates decimals	Annual mean temperature (°C)	Annual mean precipitation (mm)	Soil type (FAO soil)	Agent
Aquismon, SLP	21.6225°, -99.02°	25.2	2265.2	Lithosols	*N*. *brasiliensis*
Tamazunchale, SLP	21.2655, -98.78°	25.4	1489.7	Lithosols	*N*. *brasiliensis*
Escobedo, NL	25.8083°, -100.32°	23	479.1	Luvic kastanozems	*N*. *brasiliensis*
Matamoros, Tamps	25.8797°, -97.50°	25.8	686.1	Pellic vertisols	*N*. *brasiliensis*
Matlalpa, SLP	21.3333°, -98.83°	24.5	1936.2	Lithosols	*N*. *brasiliensis*
Apodaca, NL	25.7597°, -100.16°	22	582.8	Luvic kastanozems	*N*. *brasiliensis*
Xilitla, SLP	21.384°, -98.99°	22.7	2713.3	Lithosols	*N*. *brasiliensis*
Nuevo Laredo, Tamps	27.4863°, -99.50°	23	523	Pellic Vertisols	*A*. *madurae*
Santa Catarina, NL	25.6833°, -100.45°	21.2	373.5	Luvic kastanozems	*N*. *brasiliensis*
San Martin, SLP	21.3833°, -98.65°	23.6	1465	Calcic xerosols	*N*. *brasiliensis*
Xilitla, SLP	21.384°, -98.99°	22.7	2713.3	Lithosols	*N*. *brasiliensis*
Aramberri, NL	24.1°, -99.81°	20.4	509	Luvic kastanozems	*A*. *madurae*
Mier y Noriega, NL	23.4267°, -100.17°	19.9	512.9	Calcic xerosols	*A*. *pelletieri*
Escobedo, NL	25.8083°, -100.32°	23	479.1	Luvic kastanozems	*N*. *brasiliensis*
Piedras Negras, Coah	28.7°, -100.52°	22.5	532	Pellic vertisols	*A*. *madurae*
Axtla de Terrazas, SLP	21.4393°, -98.87°	25.2	2063.4	Eutric cambisols	*N*. *brasiliensis*
San Pedro, NL	25.6641°, -100.40°	23.3	799.3	Luvic kastanozems	*N*. *brasiliensis*
Ciudad Victoria, Tamps	23.7361°, -99.14°	23.6	737.5	Luvic phaeozems	*N*. *brasiliensis*
Matlalpa, SLP	21.3333°, -98.83°	24.5	1936.2	Lithosols	*N*. *brasiliensis*
Juarez, NL	25.6463°, -100.09°	23.3	799.3	Luvic kastanozems	*Nocardia* spp.
Tampico, Tamps	22.2552°, -97.86°	24.8	1135.8	Pellic vertisols	*N*. *brasiliensis*
Guadalupe, NL	25.6775°, -100.25°	23.9	749.5	Luvic kastanozems	*N*. *brasiliensis*
Guadalupe, NL	25.6775°, -100.25°	23.9	749.5	Luvic kastanozems	*A*. *madurae*
Galeana, NL	24.8333°, -100.06°	17.3	500.8	Lithosols	*N*. *brasiliensis*
Monterrey, NL	25.6713°, -100.30°	23.3	799.3	Luvic kastanozems	*Nocardia* spp.
Tantoyuca, Ver	21.3518°, -98.22°	23.5	1181.7	Eutric cambisols	*N*. *brasiliensis*
Ciudad Valles, SLP	21.9936°, -99.01°	24.8	1238.6	Luvic phaeozems	*N*. *brasiliensis*
Monterrey, NL	25.6713°, -100.30°	23.3	799.3	Luvic kastanozems	*N*. *brasiliensis*
Hidalgo, Mx	20.4783°, -98.86°	17.2	395.9	Eutric regosols/orthic luvisols	*N*. *brasiliensis*
San Nicolas, Tamps	24.6833, -98.81°	21.1	704.4	Rendzinas	*A*. *madurae*
Acuña, Coah	29.3241°, -100.93°	22	494.6	Calcic kastanozems	*N*. *brasiliensis*

A total of 11 (35.4%) subjects referred previous thorn trauma with *Acacia* or *Prosopis* plants including 5 (16.1%) with specific association to *S*. *greggi*, *V*. *farnesiana* or *V*. *rigidula*. A search was performed in the Global Biodiversity Information Facility database to document the presence of the genus or species of thorny plants referred (*Acacia* spp, *Prosopis* spp, *S*. *greggi*, *V*. *farnesiana*, and *V*. *rigidula*) in the states. In Nuevo León, Coahuila, San Luis Potosí, and Tamaulipas states, where 20 (64.5%) of the cases were present, these genus and species were all present; in Hidalgo, San Luis Potosí and Veracruz states where 11 (35.5%) cases were documented, *Acacia* spp., *Prosopis* spp, *V*. *farnesiana* and *V*. *rigidula* species were present. Overall, the genus and species investigated were in all states except for *S*. *greggi*, which was not present in three states (Hidalgo, San Luis Potosí, and Veracruz). The most common soil type registered was luvic kastanozems in 11 (35.5%) cases, followed by lithosols, 7 (22.6%), and pellic vertisols, 4 (12.9%). Other soils were present in only 2 cases (Fig 1).

We identified two disease cluster areas, one in Nuevo Leon, with a predominantly luvic kastanozems soil type, a mean annual temperature of 22° and a mean annual precipitation of 585.2 mm [10]; cases by *A*. *pelletieri*, *A*.*madurae*, *N*. *brasilensis* and *Nocardia* spp were identified in this area. The second cluster was in San Luis Potosí, predominantly in lithosols type soil, with a mean annual temperature of 23.5°, and a mean annual precipitation of 635.4 mm [10]; in this area, all the cases were caused by *N*. *brasiliensis*.

*A*. *madurae* cases were identified in rendzinas, luvic kastanozems, pellic vertisols, and lithosols soil, and *A*. *pelletieri* cases in calcic xerosols, luvic kastanozems, and rendzinas soil.

## Discussion

Mycetoma is different from other neglected tropical diseases because it is caused by more than 50 different species of fungi and bacteria. Nonetheless, [2, 3]most actinomycetoma and eumycetoma cases are secondary to a few species with distinct geographic distribution. Most geographic and environmental information on this subject comes from eumycetoma endemic areas, particularly Sudan, where *M*. *mycetomatis* cases predominate [4]. As North America etiological agents distinctly vary from those encountered in Asia and Africa, information regarding these regions is necessary.

Hay et al. [11] published a comprehensive review of the cartographic progress in infectious diseases and evidence-based suggestions to decide whether an infection should be mapped. Employing their suggested classification, the mycetoma recommendation was not to map the disease due to it being endemic worldwide. While mycetoma cases have been sporadically reported worldwide, it is predominantly encountered in few countries with distinct geographical, climatological, and population characteristics. Less than 15 countries have reported more than 100 cases [1, 12]. Disease mapping may help understand the yet cryptic life cycle, reservoir, and transmission routes of these microorganisms.

Samy et al. [4] mapped the risk of mycetoma by ecological niche modeling in Sudan and South Sudan. Amongst the models tested, the one employing soil type, *Acacia* distribution, normalized difference vegetation index, and land surface temperature, performed better that the other models evaluated. This report represented an important step in the effort to identify transmission risk areas.

Bonifaz et al. [2] reviewed mycetoma cases from Argentina. Santiago del Estero, a province in northern Argentina, was the region with the most cases. It has a semi-arid climate with an average temperature of 27°C and 400 to 600 mm of annual rainfall. Most cases referred previous trauma with thorns from *P*. *ruscifolia*, a native tree with big spines locally known as “viñal”. *A*. *madurae* and *T*. *grisae* were the most commonly etiological agents isolated in that region. In Tucum and Salta, two Northern provinces of Argentina, the most prevalent microorganism was *N*. *brasiliensis*. Tucum average temperature is 20°C with 900 to 1000 mm of annual rainfall, while Salta averages 17°C with 690 mm of annual rainfall.

As previously mentioned, thorn and splinter prick wounds of different species have been associated with mycetoma suggesting a relationship between the casual agents and the plants [2]. Patients with mycetoma often work in agropastoral areas walking barefooted or using sandals, being susceptible to a traumatic lesion with thorn or splinters [3, 13, 14]. Even though in Mexico is common that people in rural areas wear sandals, all our patients wore boots and shoes. *Acacia* thorns have been found embedded in mycetoma lesions during surgery [15] and these trees and mycetoma cases appeared to overlap in central Sudan while using ecological niche modeling although not specifying precisely which species [4]. Other plants that have been associated include *P*. *ruscifolia* and *P*. *juliflora* which belong to the subfamily *Mimosoideae* [2, 16]. In our series, the most commonly referred species and genus were *V*. *farnesiana*, known locally as *huizache*; *V*. *rigidula*, known as C*haparro prieto*; *S*. *greggii*, also named C*atclaw*; *Prosopis* spp. known as M*esquite*; and the *Agave* genus. Most of these plants were encountered in the states where the cases were documented. A future direction would be to map the geographical distribution of these plants with the mycetoma cases for environmental niche modelling.

Analysis of soil types and case mapping may help direct isolation from the environmental efforts [17, 18]. The three most frequent soil types identified in our study were luvic kastanozems, lithosols, and vertisols. Luvic kastanozems are dry soils rich in humus composed of a brown topsoil around 40 cm thick. It has a pH of 8, low water storage capacity, and high organic matter content. It is present in close to 465 million hectares of the earth´s surface and predominates in steppe regions with an annual precipitation of 200–400 mm [19]. It is also present in Tucuman (Argentina) and Morocco where *N*. *brasiliensis* cases predominate just as in Northeast Mexico [3].

The second most frequent soil is lithosols which occupies 28.3% of all soils in Mexico [20]. It is formed by a thin rocky soil with great quantities of calcium carbonate in form of limestone, distributed in 260 million hectares worldwide [21]; it has low permeability and predominates in highly eroded areas [20]. It can be found in Senegal, Argentina and Morocco, where cases due to actinomycetes are predominant [12].

The third most frequent soil was vertisols, which covers around 316 million km^2^ world-wide [22]. It is found in semi-arid areas with an average annual rainfall between 500–1000 mm. Additionally, it is rich in expansive clay that has wide cracks that open and close periodically varying with moisture [19]. It is also present in other endemic mycetoma areas, such as Sudan, India, Senegal, Argentina, and Venezuela [23].

Mycetoma cases by *A*. *madurae* are prevalent in Morocco and Argentina, places that share lithosols, kastanozems, vertisols, and rendzinas soil types [12, 22]; in our study, five cases of *A*. *madurae* referred to places with those soil types except for lithosols.

Strengths of the present report include species confirmation by molecular techniques of cultured strains, the precise georeferencing of cases, and the inclusion of environmental variables. Additionally, it is one of the few actinomycetoma reports that maps mycetoma cases and describes biophysical characteristics of the region where the cases occurred.

Our study has many limitations. Our study design was single-center with a small sample size, and descriptive which limits its conclusions. Nonetheless, it may serve as a first attempt to map actinomycetoma cases in America which is one of the knowledge gaps of this neglected tropical disease. Multicentric studies using ecological niche modelling are necessary to give a better overview of the disease and establish prediction models. Furthermore, more research regarding soil and vegetation analyzes is necessary to direct efforts of isolating the etiological agents of mycetoma. Our study does not have the necessary information to associate vegetation contact with disease development. Contrary to eumycetoma, information about actinomycetoma relationship with certain vegetation is scarce. Our study helps to identify certain vegetation that may have a link to the disease. More studies with a quantitative analysis are needed to confirm this. Another limitation is that we did not have data about contact with animals or animal dung, or the specific type of trauma.

In conclusion, mycetoma cases in Northeast Mexico occurred in places with a mean annual temperature ranging between 17.2°C and 25.8°C, with a variable mean annual precipitation between 373.5 mm to 2713.3 mm. Most cases occurred in places with a kastanozems and lithosols soil type. We believe our information is useful as a first initial glance to direct efforts regarding isolation of etiological agents in nature, developing public health strategies in the endemic areas, and future ecological niche models. Further studies with a larger number of cases from referral centers will help develop environmental niche modelling for these actinomycetes and fungi.

## Supporting information

S1 Checklist STROBE checklist. (DOCX)

S1 Data (XLSX)
